# Serotonin, insulin, leptin and glycolipid metabolic factor’s relationship in obesity

**DOI:** 10.1192/j.eurpsy.2023.908

**Published:** 2023-07-19

**Authors:** S. Palermo, I. Chiarantini, G. Cappellato, A. Arone, L. Massoni, S. Fantasia, M. Violi, D. Marazziti

**Affiliations:** Aoup, Pisa, Italy

## Abstract

**Introduction:**

Obesity, defined by an excessive body fat accumulation, is a non-communicable condition attaining epidemic proportions in economically developed countries.

**Objectives:**

To provide evidence to the link between serotonin (5-HT), energy metabolism and the human obese phenotype, the present study investigated the binding and function of the platelet 5-HT transporter (SERT), in relation to circulating insulin, leptin, glycolipid metabolic parameters and body-mass indices (BMIs, Kg m^-2^).

**Methods:**

The study included an observational clinical cohort of 74 drug-free subjects (51W; 23M), recruited on the basis of divergent BMIs (16.5-54.8 Kg m^-2^). All subjects were tested for their blood glycolipid profile together platelet [^3^H]-paroxetine ([^3^H]-Par) binding and [^3^H]-5-HT reuptake measurements from April 1^st^ to June 30^th^ 2019.

**Results:**

The [^3^H]-Par B_max_ (fmol/mg proteins) was progressively reduced with increasing BMIs (p<.001), without changes in affinity. Moreover, B_max_ was negatively correlated with BMI, waist/hip circumferences, triglycerides, glucose, insulin and leptin, while positively with HDL cholesterol (p<.01). The reduction of 5-HT uptake rate (V_max_, pmol//min/10^9^platelets) amongst BMI groups was not statistically significant, but V_max_ negatively correlated with leptin and uptake affinity values (p<.05). Besides, [^3^H]-Par affinity values positively correlated with glycaemia and triglycerides, while [^3^H]-5-HT reuptake affinity with glycaemia only (p<.05). Finally, these correlations were specific of obese subjects, while, from multivariate linear-regression analysis conducted on all subjects, insulin (p=.006) resulting negatively related to B_max_ independently from BMI.

**Conclusions:**

The present findings would suggest the presence of a dysfunctional insulin/5-HT/leptin axis in obesity, differentially impinging the density, function and/or affinity of the platelet SERT, as the result of complex appetite/reward-related interactions between the brain, gut, pancreatic islets and adipose tissue. In addition, they support the foremost cooperation of insulin and 5-HT in maintaining energy homeostasis.

**Disclosure of Interest:**

None Declared

